# Oxytocin receptor binding in the titi monkey hippocampal formation is associated with parental status and partner affiliation

**DOI:** 10.1038/s41598-020-74243-1

**Published:** 2020-10-14

**Authors:** Alexander Baxter, M. Anderson, A. M. Seelke, E. L. Kinnally, S. M. Freeman, K. L. Bales

**Affiliations:** 1 California National Primate Research Center, University of California, Davis, One Shields Ave, Davis, CA 95616 USA; 2 Department of Psychology, University of California, Davis, Davis, USA; 3 Department of Biology, Utah State University, Logan, USA; 4 Department of Neurobiology, Physiology, and Behavior, University of California, Davis, Davis, USA

**Keywords:** Neuroscience, Social behaviour, Social neuroscience, Transporters in the nervous system

## Abstract

Social cognition is facilitated by oxytocin receptors (OXTR) in the hippocampus, a brain region that changes dynamically with pregnancy, parturition, and parenting experience. We investigated the impact of parenthood on hippocampal OXTR in male and female titi monkeys, a pair-bonding primate species that exhibits biparental care of offspring. We hypothesized that in postmortem brain tissue, OXTR binding in the hippocampal formation would differ between parents and non-parents, and that OXTR density would correlate with frequencies of observed parenting and affiliative behaviors between partners. Subjects were 10 adult titi monkeys. OXTR binding in the hippocampus (CA1, CA2/3, CA4, dentate gyrus, subiculum) and presubiculum layers (PSB1, PSB3) was determined using receptor autoradiography. The average frequency of partner affiliation (*Proximity*, *Contact*, and *Tail Twining*) and infant carrying were determined from longitudinal observations (5–6 per day). Analyses showed that parents exhibited higher OXTR binding than non-parents in PSB1 (*t*_(8)_ = − 2.33, *p* = 0.048), and that OXTR binding in the total presubiculm correlated negatively with *Proximity* (*r* = − 0.88) and *Contact* (*r* = − 0.91), but not *Tail Twining* or infant carrying. These results suggest that OXTR binding in the presubiculum supports pair bonding and parenting behavior, potentially by mediating changes in hippocampal plasticity.

## Introduction

The transition to parenthood brings many changes in neural physiology and neural plasticity. For many mammals, these changes in the brain are necessary to enable parental behavior. For example, although virgin female rats typically avoid or attack pups, changes occur in the maternal brain after they give birth to their first litter that increase approach behavior and inhibit avoidance behavior, leading to maternal care of offspring (see^1^). This pathway is well-characterized and is regulated by the hormones oxytocin, estrogen, and progesterone acting in the medial preoptic area, which leads to top-down modulation of mesolimbic dopaminergic structures, including the nucleus accumbens shell, ventral tegmental area, and ventral pallidum^1–3^. These changes in reward- and motivation-related circuits enable the onset of maternal behavior in rodents^1^, and similar brain changes occur in other mammals when individuals become parents^1,2^. Parenthood is also associated with changes in many other parts of the brain, particularly regions involved in cognition^4^, emotion, and affect^5^. In some cases, these changes can be semi-permanent, with changes in connectivity and plasticity lasting beyond the time when offspring are being actively cared for^6–8^.

The hippocampus is one such structure that plays an important role in parenting behavior and exhibits drastic changes in plasticity when individuals become parents^4,8,9^. Research in rodent mothers has shown that many changes occur in hippocampal plasticity and dendritic architecture during pregnancy, the post-partum period, and weaning, an effect that coincides with behavioral changes in spatial navigation (see^8^). Some studies suggest that these effects last throughout the rodent’s life, even after offspring have dispersed and are no longer being cared for, and that the effects are cumulative with subsequent litters (however, evidence is mixed; for a comprehensive review, see^8^). Various changes in hippocampal plasticity also occur in humans and other animal species during pregnancy and the transition to parenthood (see^4,8,9^). For example, using functional magnetic resonance imaging, Hoekzema, et al.^10^ found that pregnant women exhibited lower hippocampal grey matter compared to non-pregnant women, and that this difference persisted even at a 2-year follow up. Women also show many changes in cognitive abilities during pregnancy, some of which persist post-partum (however, it should be noted that post-partum cognitive changes have not been studied as extensively as those that occur during pregnancy, and evidence is mixed; see^11–14^). Although the role of the hippocampus in paternal care has not been studied as extensively as it has in maternal care, there is some evidence that the hippocampus contributes to fathering behavior, as well as to the general neurocircuitry underlying attachment (see^15,16^).

Oxytocin is a key hormonal regulator of many of the brain regions involved in parenting and broader social attachment^3,15,17^, including the hippocampus^18^. In the brain, oxytocin is produced in the paraventricular and supraoptic nuclei of the hypothalamus in response to different types of social and tactile stimuli and exerts its neuro-modulatory effects in various brain regions by binding to the oxytocin receptor (OXTR), a G-protein-coupled receptor^19^. In the hippocampus, oxytocin signaling plays an important role in regulating cognitive functions in certain social contexts^18^. For example, in rodents, OXTR binding is necessary for discriminating between different social (but not non-social) stimuli^20^. Oxytocin plays an important role in regulating neuronal firing and neurogenesis in the hippocampus, and in some circumstances, oxytocin can inhibit or facilitate long-term potentiation (see^18^). It can also protect hippocampal neurons from the neurotoxic effects of cortisol^21^. Through these and other mechanisms, oxytocin plays an important role in regulating hippocampal neuroplasticity, which renders it an excellent candidate system for the neural mechanisms underlying parenting-related changes in the hippocampus^8^. In support of this premise, some rodent studies suggest that hippocampal OXTR binding changes in response to pup exposure^22^ and during pregnancy^23^. However, the evidence is mixed, and some studies have found no differences in females’ hippocampal OXTR binding at different stages of pregnancy, parturition, and the post-partum^24–26^.

**Figure 1:**
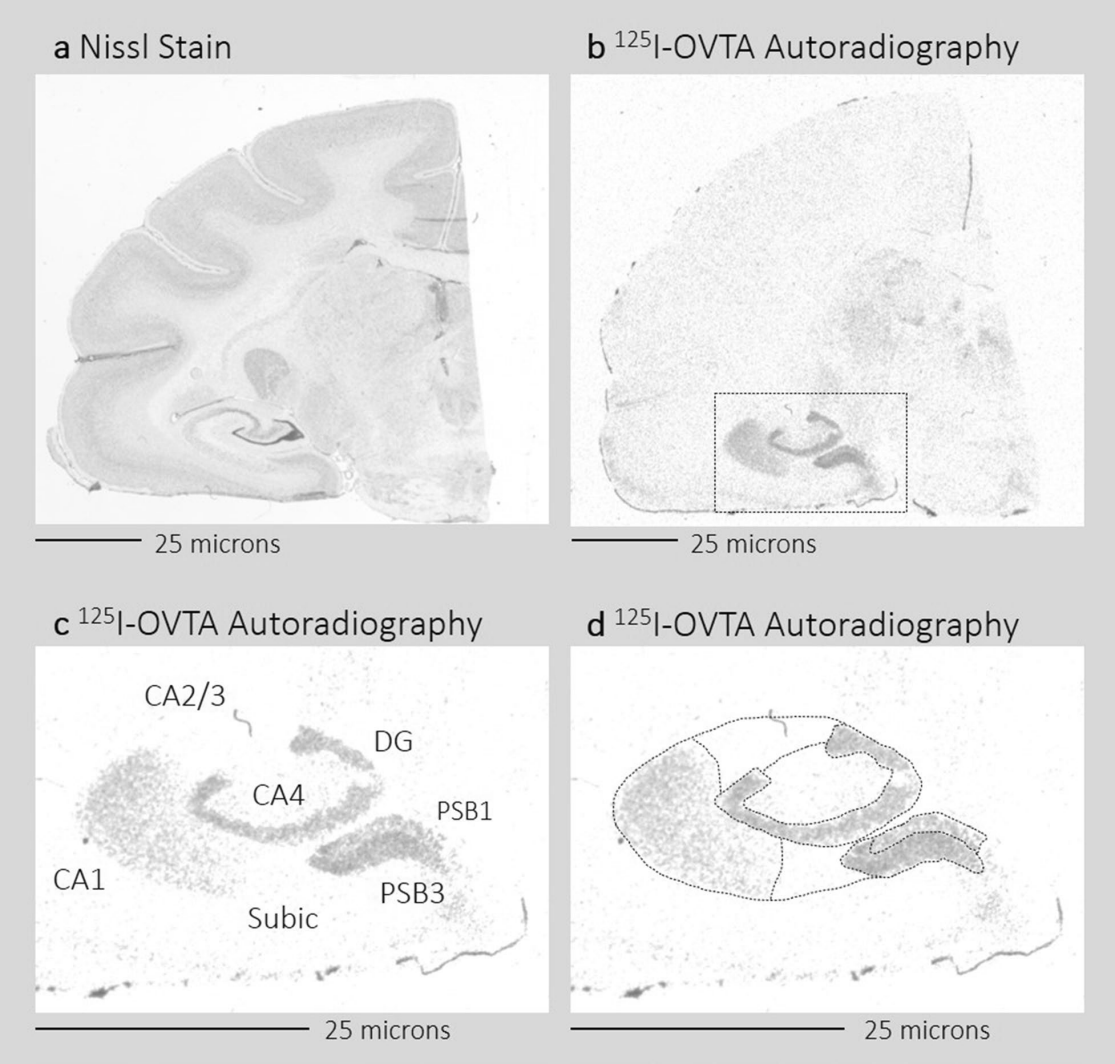
Schematic of hippocampus and presubiculum brain regions quantified. The figure shows two adjacent sections from one representative titi monkey brain. The section in (**a**) was stained using Nissl staining. The section in (**b**) underwent ^125^I-OVTA autoradiography to identify OXTR binding. (**c**,**d**) A zoomed in segment of (**b**) (the zoomed region is indicated by the black box). (**c**) The brain regions that were quantified, and (**d**) depicts the boundaries that were traced to quantify each brain region. ^*125*^*I-OVTA* ^125^I-ornithine vasotocin analog, *CA1*, *CA2/3, CA4* CA fields of the hippocampus, *DG* dentate gyrus, *Subic* subiculum, *PSB1* presubiculum layer 1, *PSB3* presubiculum layer 3.

Given that most of the research on the neurobiology of parenthood has been conducted in rodent species and relatively few studies have been conducted in primates^2^, the purpose of this study was to investigate whether hippocampal OXTR binding in the brains of titi monkey (*Plecturocebus cupreus*) differs between parents and non-parents. Titi monkeys are a biparental and socially monogamous species and are therefore an ideal nonhuman primate model for investigating the neurobiology of parenting and pair-bonding^27^. We investigated whether OXTR binding density in the hippocampal formation was associated with parental status and parenting behavior. Recently, Freeman, et al.^28^ characterized the distribution of OXTR binding sites in the titi monkey brain and found dense OXTR in the dentate gyrus and presubiculum, a medial temporal lobe structure that supports hippocampus functioning^29^. In the current study, we build on this work with a larger sample of titi monkey post-mortem brain tissue and used the same receptor autoradiography methods to investigate OXTR binding throughout the hippocampal formation (including the CA1, CA2/3, and CA4 fields, the dentate gyrus, and the subiculum) and the presubiculum [including layers 1 (PSB1) and 3 (PSB3)] (see Fig. 1). We explored whether OXTR binding in these hippocampal regions differed between parents (monkeys with experience rearing offspring) and non-parents (monkeys with no experience rearing offspring). We hypothesized that parents would exhibit higher OXTR binding than non-parents. We also investigated whether OXTR binding was associated with individual differences in the average amount of time that parents spent carrying their infants, a species-typical parenting behavior^30,31^. To do this, we utilized a large archive of longitudinal daily observations that were conducted while the subjects were still alive, spanning between 2–9 months of observations per parent (see Supplementary Table S1). We hypothesized that higher OXTR binding density in post-mortem brain tissue would predict higher levels of infant carrying.

We also investigated two secondary research questions. First, we investigated whether OXTR binding in the hippocampus and presubiculum was associated with affiliative behavior between pair mates. Oxytocin plays a vital role in regulating pair bonding^32^, and in humans, it is linked with romantic bonding^33,34^. For example, one study of people in new romantic relationships showed that plasma oxytocin, measured within the first 3 months of dating, predicted which couples stayed together and which separated 6 months later^33^. This study further showed that plasma oxytocin levels were correlated with levels of dyadic synchrony, physical touching, and positive affect among new romantic partners^33^. In both male^35^ and female^36,37^ prairie voles, increased OXTR expression in the nucleus accumbens is associated with an accelerated formation of a partner preference (relative to a preference for an opposite-sex stranger), a hallmark characteristic of a pair bond. To our knowledge, the relationship between hippocampal OXTR binding and affiliative behavior with a pair mate has not been explored in non-human primates. Utilizing a large archive of longitudinal observations spanning between 5–21 months of data for each subject (see Supplementary Table S1), we investigated whether hippocampal OXTR binding in post-mortem brain tissue was associated with the average amount of time that titi monkeys spent affiliating with their partner. We hypothesized that OXTR binding would correlate positively with species-typical^27^ affiliative behaviors with the partner, including *Proximity*, *Contact*, and *Tail Twining*.

Second, we investigated whether OXTR binding in the hippocampal formation differed between males and females. The oxytocin system is responsive to sex hormones (see, for example^38–40^), and sex differences have been reported in both rodents and primates across many peripheral and central oxytocin measures (for a comprehensive review, see^41^). Thus far, sex differences in hippocampal OXTR binding have been studied primarily in rodents, and research has yielded mixed evidence. For example, although seven studies (across six different rodent species) failed to find sex differences in hippocampal OXTR binding^22,42–47^, two studies (across three rodent species) found that females exhibited higher hippocampal OXTR binding than males^48,49^, and two other studies (across two rodent species) found that males exhibited higher hippocampal OXTR binding than females^50,51^. In humans, two studies investigated OXTR binding in the basal forebrain and midbrain in post-mortem brain tissue, and neither found sex differences in any of the regions that were studied^52,53^. However, these human studies did not investigate hippocampal OXTR binding, and further investigation is needed in primate species. We hypothesized that titi monkeys would exhibit sex differences in hippocampal OXTR binding.

## Results

**Figure 2:**
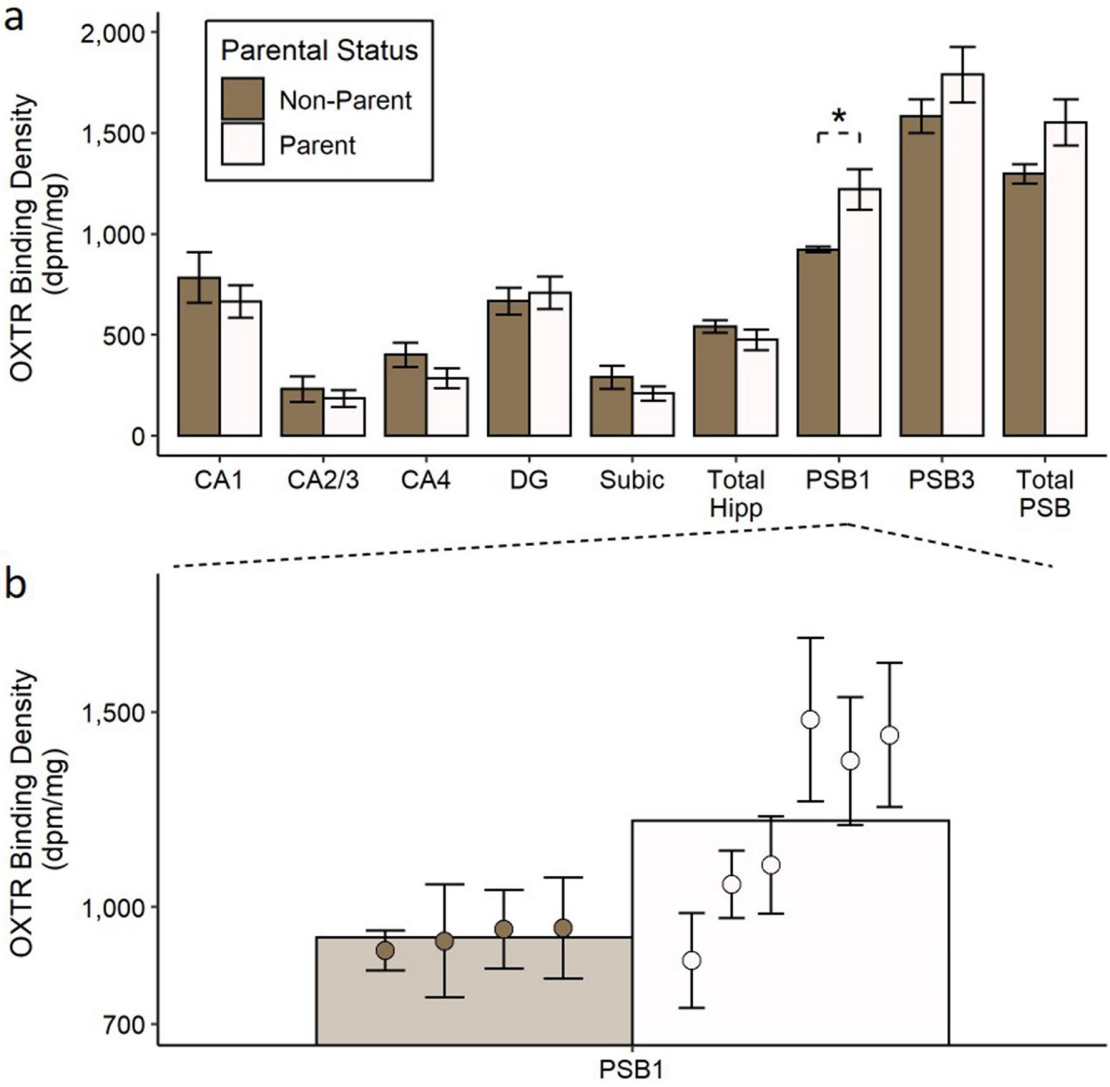
Parents exhibit higher OXTR binding density in PSB1. The top panel (**a**) shows the average OXTR binding density for parents and non-parents in each brain region quantified, and error bars indicate ± 1 standard error. *The group comparison is significant at *p* < 0.05. The bottom panel (**b**) shows a zoomed view of the average OXTR binding density in PSB1 for parents and non-parents (bars), with the average OXTR binding for each subject (circles) overlaid on top. The error bars in this panel correspond to ± 1 standard error across the different sections that were averaged for each subject. *dpm* disintegrations per minute, *CA1*, *CA2/3, CA4* CA fields of the hippocampus, *DG* dentate gyrus, *Subic* subiculum, *Total Hipp* total hippocampus area (including CA1, CA2/3, CA4, DG, and Subic) , *PSB1* presubiculum layer 1, *PSB3* presubiculum layer 3, *total presubiculum* total presubiculum area (including PSB1 and PSB3).

A MANOVA showed a main effect of parental status on OXTR binding density across PSB1 and PSB3 (*F*_(1, 8)_ = 5.70, *p* = 0.034, Pillai’s trace = 0.62). Follow up t-tests showed that this effect was specific to PSB1 (*t*_(8)_ =  − 2.35, *p* = 0.047, *d* = − 1.52; see Fig. 2), with no significant differences between parents and non-parents in PSB3 (*t*_(8)_ = − 1.12, *p* = 0.29, *d* = − 0.73). There was no effect of parental status on OXTR binding across the total presubiculum (i.e., when OXTR binding was quantified across PSB1 and PSB3 together; *t*_(8)_ =  − 1.72, *p* = 0.12, *d* = − 1.11), and there was no effect of parental status in a MANOVA with OXTR binding in the hippocampus subregions (CA1, CA2/3, CA4, dentate gyrus, and the subiculum) as dependent variables (*F*_(1, 8)_ = 0.78, *p* = 0.61, Pillai’s trace = 0.49). There was no significant difference between parents and non-parents in total hippocampus OXTR binding (i.e., when OXTR binding was quantified across the hippocampus subregions together; *t*_(8)_ = 0.95, *p* = 0.37, *d* = 0.62).

**Figure 3:**
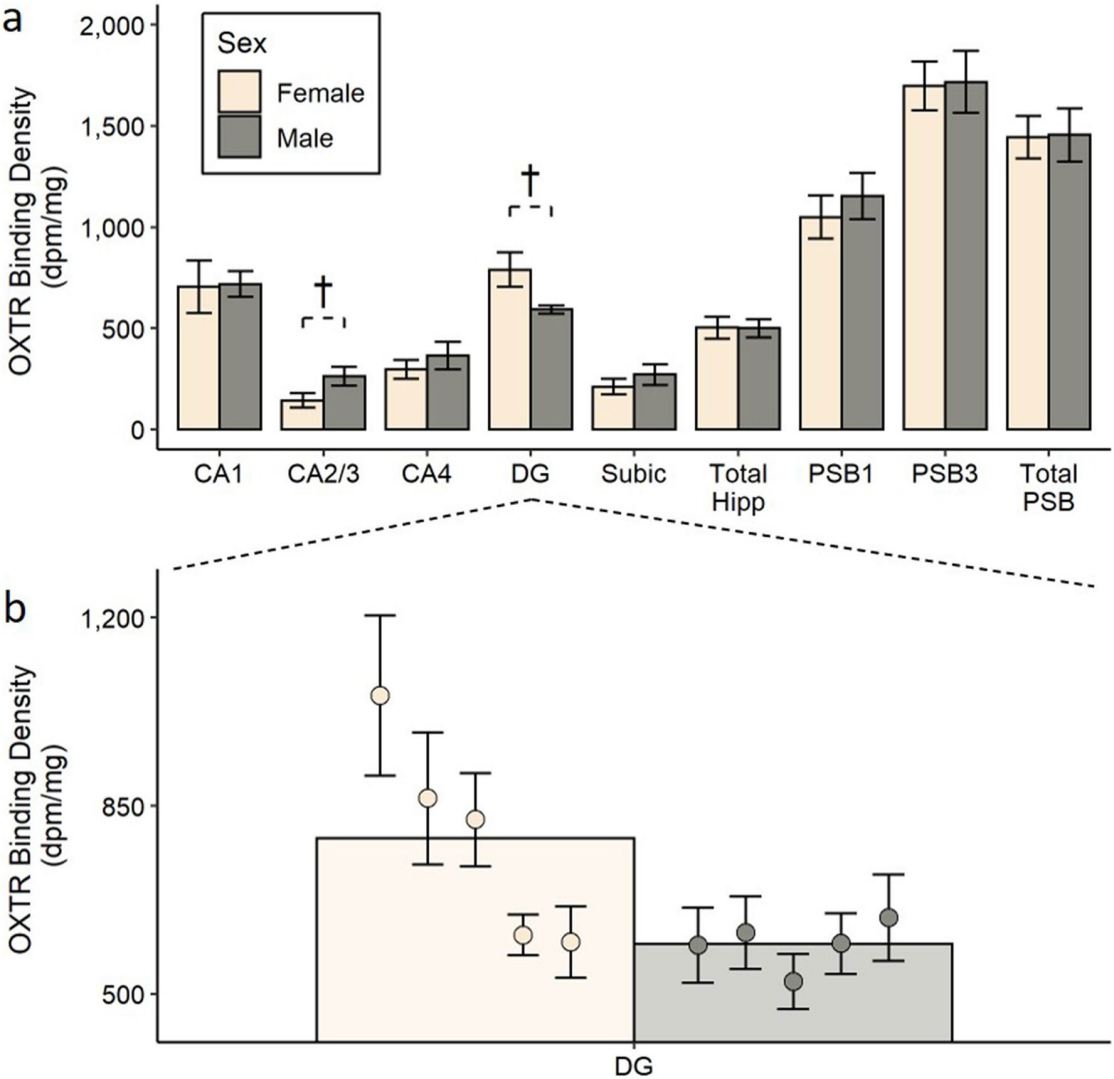
No sex differences in OXTR binding in the hippocampus or presubiculum. The top panel (**a**) shows the average OXTR binding density for males and females in each brain region quantified, and error bars indicate ± 1 standard error. †The *p* value for the group comparison is < 0.10. The bottom panel (**b**) shows a zoomed view of the average OXTR binding density in the dentate gyrus for males and females (bars), with the average OXTR binding for each subject (circles) overlaid on top. The error bars in this panel correspond to the ± 1 standard error across the different sections that were averaged for each subject. *dpm* disintegrations per minute, *CA1*, *CA2/3, CA4* CA fields of the hippocampus, *DG* dentate gyrus, *Subic* subiculum, *Total Hipp* total hippocampus area (including CA1, CA2/3, CA4, DG, and Subic) , *PSB1* presubiculum layer 1, *PSB3* presubiculum layer 3, *Tot. PSB* total presubiculum area (including PSB1 and PSB3).

There were no significant sex differences in OXTR binding in a MANOVA with OXTR binding in PSB1 and PSB3 as dependent variables (*F*_(1, 8)_ = 0.87, *p* = 0.46, Pillai’s trace = 0.20) or in a MANOVA with OXTR binding in the hippocampus subregions (CA1, CA2/3, CA4, dentate gyrus, and the subiculum) as dependent variables (*F*_(1, 8)_ = 5.26, *p* = 0.066, Pillai’s trace = 0.87). Although the sex difference across the hippocampus subregions did not achieve formal statistical significance (*p* = 0.066), because sex differences were hypothesized, exploratory t-tests were performed to investigate this effect across the different hippocampus brain regions. There was a trending sex difference in OXTR binding in the dentate gyrus (*t*_(8)_ = 2.24, *p* = 0.055, *d* = 1.42; see Fig. 3) and in CA2/3 (*t*_(8)_ =  − 2.06, *p* = 0.073, *d* = − 1.30). There were no sex differences in OXTR binding in the other hippocampus subregions investigated, nor in the total hippocampus (*t*_(8)_ = 0.05, *p* = 0.96, *d* = 0.03) or total presubiculum (*t*_(8)_ = − 0.07, *p* = 0.95, *d* = − 0.04).

**Figure 4:**
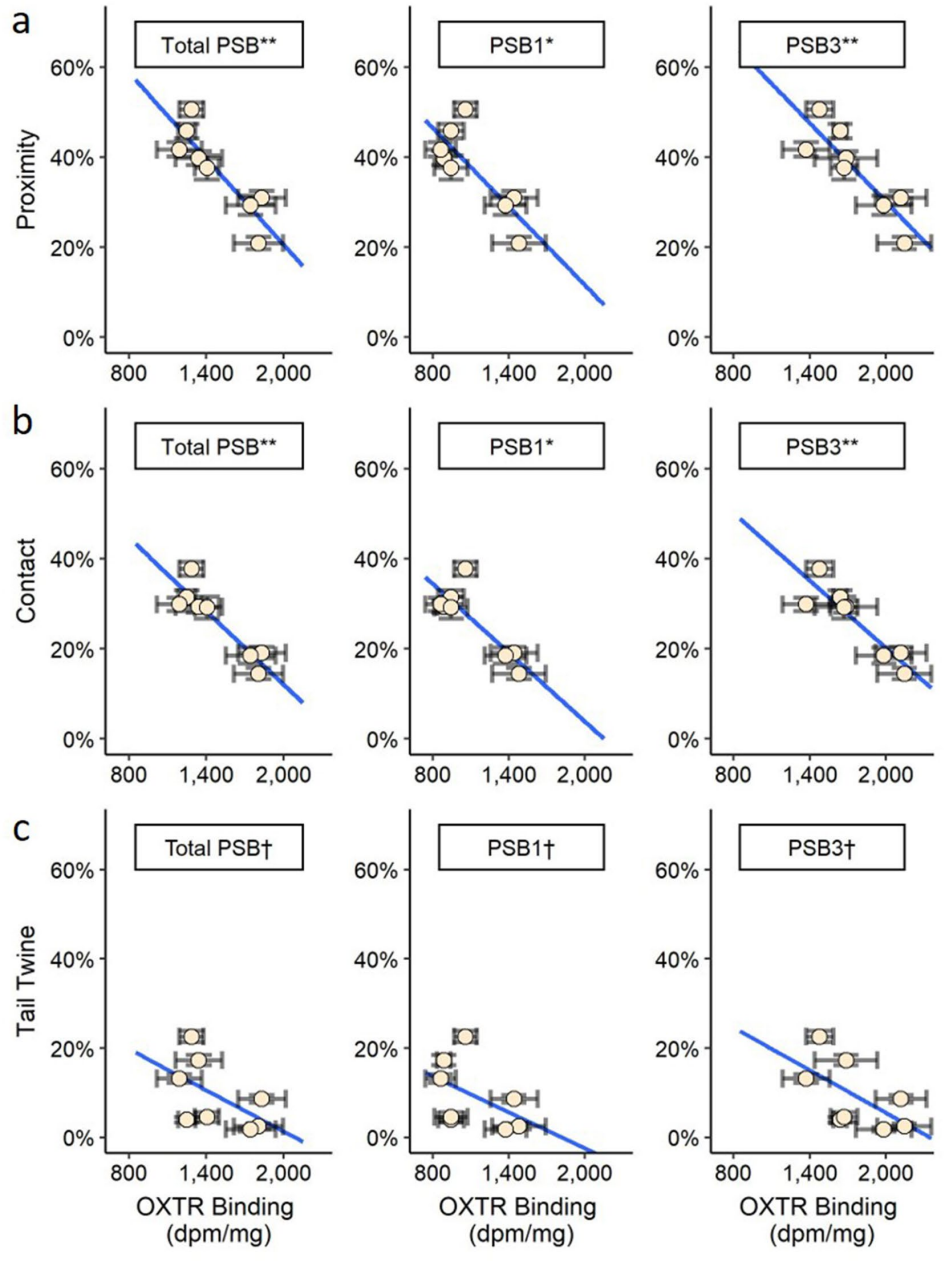
OXTR binding in the presubiculum is associated with affiliative behavior with the pair mate. The figure shows the associations between average OXTR binding density (in the total presubiculum, PSB1, and PSB3) and average *Proximity* (**a**), *Contact* (**b**), and *Tail Twining* (**c**). The vertical error bars represent ± 1 standard error across the affiliation data averaged for the subject, and the horizontal errors bars represent ± 1 standard error across the OXTR binding of each brain section averaged for the subject. *The linear association between OXTR binding and the average affiliation state was significant at *p* < 0.05, and remained significant when pair tenure was controlled; **the linear association between OXTR binding and the average affiliation state was significant at *p* < 0.05 and remained significant when analyzed using Spearman rho rank correlation and when pair tenure was controlled; †the linear association between OXTR binding was not significant (*p* ranged from 0.11 to 0.25). For a full summary of the analyses, see Table 2. *dpm* disintegrations per minute, *PSB1* presubiculum layer 1, *PSB3* presubiculum layer 3, *Total presubiculum* total presubiculum area (including PSB1 and PSB3).

Pearson correlation analyses revealed that OXTR binding in PSB1, PSB3, and the total presubiculum were correlated negatively with average *Proximity* and *Contact* (Pearson *r* ranged from − 0.84 to − 0.91, *p* < 0.01; see Table 1; Fig. 4). These associations remained significant even when analyses were repeated using multiple regression to control for pair tenure (the amount of time that subjects had already been paired together when affiliation data began to be collected; see Table 2). When analyses were repeated using Spearman rho correlation, four associations remained significant (see Table 2), including the associations between *Proximity* and OXTR binding in the total presubiculum (ρ = − 0.83, *p* = 0.01), *Contact* and OXTR binding in the total presubiculum (ρ  = − 0.83, *p* = 0.01), *Proximity* and OXTR binding in PSB3 (ρ  = − 0.88, *p* = 0.004), and *Contact* and OXTR binding density in PSB3 (ρ  = − 0.88, *p* = 0.004); the associations between OXTR binding in PSB1 and *Proximity* and *Contact* were marginally significant (*p* = 0.071; see Table 2). No correlations between OXTR binding in the presubiculum and average *Tail Twining* were significant in any of the analyses performed, although the associations were in the same direction as the other affiliative behaviors (see Table 2). No other associations between average partner affiliation and OXTR binding in any other brain regions outside the presubiculum were significant, and there were no significant associations between the average amount of infant carrying and OXTR binding in any brain region (*p* > 0.32; see Table 1).

**Table 1:** Correlation matrix of analyzed variables.

	Tot. Hip	CA1	CA2/3	CA4	DG	Subic	Tot. PSB	PSB1	PSB3	P	C	TT	Carry	Age	Weight
**Hippocampus**															
Tot. Hip	–														
CA1	0.73*	–													
CA2/3	0.68*	0.32	–												
CA4	0.76*	0.37	0.88*	-											
DG	0.41	− 0.02	− 0.09	0.05	–										
Subic	0.68*	0.43	0.85*	0.93*	− 0.15	–									
**Presubiculum**															
Tot. PSB	0.54	0.46	0.34	0.22	0.30	0.16	–								
PSB1	0.42	0.34	0.39	0.18	0.17	0.15	0.96*	–							
PSB3	0.69*	0.55†	0.48	0.36	0.33	0.31	0.96*	0.90*	–						
**Affiliation states**															
Proximity	− 0.49	− 0.41	− 0.31	− 0.25	− 0.21	− 0.08	− 0.88*	− 0.80*	− 0.88*	–					
Contact	− 0.53	− 0.35	− 0.40	− 0.36	− 0.28	− 0.17	− 0.91*	− 0.84*	− 0.91*	0.98*	–				
Tail twine	− 0.58	− 0.62	− 0.54	− 0.63†	0.14	− 0.58	− 0.54	− 0.46	− 0.60	0.67†	0.69†	–			
**Infant carrying**															
Carrying	− 0.32	− 0.01	0.03	− 0.34	− 0.49	− 0.47	− 0.13	− 0.07	− 0.10	0.01	0.11	− 0.11	–		
**Covariates**															
Age	0.12	0.29	0.53	0.16	− 0.54	0.29	0.01	0.18	0.11	0.05	0.07	− 0.13	0.62	–	
Weight	− 0.02	− 0.08	0.38	0.18	− 0.27	0.06	− 0.28	− 0.15	− 0.23	0.23	0.18	0.07	0.89*	0.61†	-
Pair tenure	− 0.02	− 0.31	− 0.17	− 0.33	0.62†	− 0.33	− 0.01	0.02	0.03	0.33	0.29	0.79*	− 0.33	− 0.07	− 0.08

The correlation table shows the Pearson correlations between average OXTR binding density (in each subregion), average pair mate affiliation states, and average infant carrying. There were 10 subjects used in correlations between OXTR binding in different brain regions, eight subjects used in correlations between OXTR binding and pair mate affiliation, and six subjects used in correlations between OXTR binding and infant carrying. * indicates *p* < 0.05; † indicates *p* < 0.10.

*CA1, CA2/3, CA4* CA fields of the hippocampus, *DG* dentate gyrus, *Subic* subiculum, *Tot. Hip*. total hippocampus area (including CA1, CA2/3, CA4, DG, and Subic) , *PSB1* presubiculum layer 1, *PSB3* presubiculum layer 3, *Tot. PSB* total presubiculum area (including PSB1 and PSB3), *P* proximity, *C* contact, *TT* tail twine, *Carry* infant carrying.

**Table 2:** Summary of associations between OXTR binding in the PSB and average pair mate affiliation states.

Affiliation measure and brain region	Pearson correlation	Spearman rank correlation	Linear regression (affiliation ~ OXTR + pair tenure)	
	*r*	ρ	_βOXTR_	_βPair Tenure_
**Proximity**				
Tot. PSB	− 0.88*	− 0.83*	− 0.76*	0.26
PSB1	− 0.80*	− 0.67†	− 0.64*	0.31
PSB3	− 0.88*	− 0.88*	− 0.79*	0.24
**Contact**				
Tot. PSB	− 0.91*	− 0.83*	− 0.80*	0.21
PSB1	− 0.84*	− 0.67†	− 0.71*	0.25
PSB3	− 0.91*	− 0.88*	− 0.84*	0.18
**Tail twine**				
Tot. PSB	− 0.54	− 0.43	− 0.28	0.75*
PSB1	− 0.46	− 0.48	− 0.21	0.78*
PSB3	− 0.60	− 0.55	− 0.35	0.72*

The table shows the associations between OXTR binding in PSB1, PSB3, and the total presubiculum with averaged pair mate *Proximity*, *Contact*, and *Tail Twining*. There were eight subjects with available data for these analyses. The first column contains the Pearson *r* correlation between the average affiliation state and the average OXTR binding density. The second column contains the Spearman rho correlation coefficient. The third and fourth columns contain the beta estimates for a linear regression model in which the average affiliation state was predicted by average OXTR binding when pair tenure (the amount of time the subject had been with its pair mate when affiliation data first began to be collected) was controlled. *p < 0.05; ^†^*p* < 0.10.

*PSB1* presubiculum layer 1, *PSB3* presubiculum layer 3, *Tot. PSB* total presubiculum area (including PSB1 and PSB3).

## Discussion

In this study we investigated whether OXTR binding in the titi monkey hippocampal formation correlated with parental status and parenting behavior. As hypothesized, we found that titi monkey parents exhibited higher OXTR binding density than non-parents in PSB1. To our knowledge, this is the first primate study to show a difference in hippocampal OXTR binding between individuals with and without parenting experience. We also investigated whether hippocampal OXTR binding was associated with individual variation in parenting behavior and partner affiliation, and we found an association between OXTR binding and the average percent of observations that monkeys were in proximity to or in contact with their partner, but only in the presubiculum. To our knowledge, this is the first report of a correlation between OXTR binding density in the hippocampal formation and variation in social behavior in a non-human primate species. There was no association between OXTR binding in any region examined and infant carrying or tail twining with the pair mate. Although we hypothesized that there would be sex differences in OXTR binding, we found only limited evidence to support this hypothesis. Together, the results of this study suggest that OXTR binding in the presubiculum may play a role in parenting and pair-bonding behavior.

In this study, we found that monkeys with parenting experience exhibited higher levels of OXTR binding in PSB1 compared to monkeys without parenting experience. Our finding suggests that the experience of rearing offspring was associated with higher levels of OXTR binding in PSB1. We speculate that parenting experience had a causal effect on increasing PSB1 OXTR binding. Congruent with this interpretation, rodent studies have shown that changes in hippocampal plasticity and related cognitive abilities occur when individuals become parents^4,7,8^. These changes in hippocampal plasticity may be moderated, in part, by hippocampal OXTR binding, which has been shown to facilitate social learning and induce changes in plasticity in response to social stimuli^18,20^, and which may also be increased during pregnancy^23^ (however, see^24–26^). Although the role of OXTR binding in the presubiculum has not been directly explored, the presubiculum is part of the extended hippocampal formation^54,55^ and supports many of the learning and memory functions regulated by the hippocampus^29^, especially in layer 1 and layer 2 of the presubiculum^55^. It also serves as a relay between the hippocampus and other cortical sensory-processing brain regions^56,57^. To the extent that OXTR binding in the presubiculum and hippocampus play similar roles in regulating plasticity and social cognition^18,20^, our finding suggests that increased OXTR binding in the hippocampal formation is a potential mechanism underlying the changes in neural plasticity that accompany parenthood. However, in the absence of cognition or plasticity data in this study, further investigation is needed to explore this hypothesis.

Although there is support for parenting experience having a causal effect in this study (see above), there may be other possible explanations for why parents exhibited higher OXTR binding in PSB1 than non-parents. One alternative interpretation is that differences in OXTR binding were pre-existing and affected which monkeys had surviving offspring and which ones did not. In this study, three of the four non-parents (including both female non-parents) previously produced a non-surviving offspring (see Supplementary Table S1), but these subjects were not considered parents for the purposes of this study because they did not have experience rearing offspring (offspring were either born dead or died on the first day of birth). Considering that OXTR binding is linked with variation in maternal and alloparental care in rodents^37,58,59^, it is possible that pre-existing low levels of OXTR binding in the non-parent group increased the likelihood that they would reject their offspring or have other birth complications that resulted in the offspring’s death. However, we believe this interpretation is unlikely, as two of the six subjects that were parents had also produced non-surviving offspring (i.e., in addition to producing a surviving offspring; see Supplementary Table S1), and preliminary analyses showed no effects of producing a non-surviving offspring on OXTR binding.

It could also be argued that the results of the study were confounded by the processes associated with producing a non-surviving offspring, including pregnancy, parturition, and/or brief exposure to an offspring, which could all potentially affect hippocampal plasticity (see, for example^7,8,60^). While this is plausible, we believe it is unlikely, as studies of rodents^24,25^ and rabbits^26^ have shown that individual differences in females’ hippocampal OXTR binding are stable across late gestation, parturition, and the post-partum period. There is mixed evidence concerning changes in hippocampal OXTR binding across the entire pregnancy. Although one study^23^ showed that pregnant female prairie voles had higher hippocampal OXTR binding than non-pregnant females, another study of female prairie voles^25^ (as well as two studies in rats^24^ and rabbits^26^) showed no effects of pregnancy on hippocampal OXTR binding. While it is possible that pregnancy and parturition had effects on the females in this study, given that all the females in the parent and non-parent groups experienced at least one pregnancy and parturition, any confounding effects would likely be equal across the two groups. Concerning the males, there is evidence that some of the neural and behavioral changes associated with fatherhood begin even before the birth of offspring^61^. For example, studies of monogamous vole species have shown that even before a male has produced offspring, sexual experience and cohabitation with a mate can lead to increased paternal behaviors towards unfamiliar pups^62,63^ as well as changes in central oxytocin^63^ and vasopressin^62^ fiber counts that approximate the phenotypes exhibited by fathers. However, these studies did not examine the hippocampus, and although several rodent studies have shown that hippocampal plasticity changes in fathers after their offspring are born^64–69^, it is still unknown whether paternal hippocampal plasticity or OXTR binding changes during the mate’s gestation. In this study, all the male parents and one of the two male non-parents had a pair mate that experienced pregnancy and parturition (see Supplementary Table S1). It is therefore possible that the experience of having (or not having) a pregnant pair mate contributed to differences between the two male non-parents in this study. Due to having only one male in each of these two categories, it was not possible to evaluate differences between these subjects inferentially. However, we note that neither of these subjects were outliers in any measure of OXTR binding. For this reason, we believe that parents exhibited higher PSB1 OXTR binding than non-parents due to the experience of rearing offspring, and not due to gestation or parturition effects alone. Further research is needed to confirm this interpretation.

In addition to differences between parents and non-parents, we also found that average levels of partner affiliation correlated strongly with OXTR binding in both layer 1 and layer 3 of the presubiculum, as well as in the total presubiculum. OXTR binding was negatively correlated with proximity and contact behavior with the partner. Although the association between OXTR binding in PSB1 and tail twining behavior was weaker and not significant, it was likewise negatively correlated with OXTR binding. The results of this study suggest that low levels of affiliative behavior are associated with high levels of OXTR binding in the hippocampal formation. This negative relationship between OXTR binding and affiliative behavior was unexpected and somewhat paradoxical, considering that studies of prairie voles^35–37^ and marmosets (*Callithrix penicillata*)^70^ suggest that increased central OXTR binding facilitates pair bonding, while OXTR blockade disrupts pair bonding^70,71^. One possible explanation for the negative correlation found in this study is that monkeys with high levels of affiliation experienced higher levels of endogenous oxytocin and/or more frequent releases of oxytocin, which potentially lead to down-regulated OXTR expression and binding in the hippocampus (see^72^). In support of this interpretation, studies in humans have shown that oxytocin is endogenously released in response to social touch^73^ and during sexual intercourse^74^, behaviors that would likely be increased in monkeys that frequently affiliate with their pair mate. Although further research is needed to explore this interpretation, particularly in the hippocampal formation, our findings nonetheless suggest that OXTR binding in the presubiculum contributes to affiliative behavior in titi monkeys, potentially by modulating certain socio-cognitive abilities that support pair bonding. For example, because the presubiculum responds to and encodes head position and orientation^75^, OXTR binding in the presubiculum may integrate stimuli and cues from the pair mate with visual attention and orienting. Further research is needed to test these hypotheses and to explore whether presubiculum OXTR binding is associated with affiliative behavior in other species.

While OXTR binding differed between parents and non-parents, it was not associated with individual differences in infant carrying behavior. While this may indicate that OXTR binding is not associated with variation in parenting behavior in titi monkeys, the null association may also be due to a small sample size, as infant carrying data were only available for the six subjects that were parents (see Supplementary Table S1). Given that several studies of prairie voles have shown associations between and maternal or alloparental behavior and OXTR binding in other parts of the brain^37,58,59,71^, we believe the latter explanation is more likely. Moreover, an analysis of infant carrying combined across titi monkey fathers and mothers may not be appropriate, as titi monkey fathers are primarily responsible for carrying infants, and in most cases mothers do not carry infants on their back (but see^27^). With only three male and three female parents, separate analyses by sex were not possible. In the future, studies with a larger sample size may be better powered to investigate whether hippocampal OXTR binding associates with differences in infant care in titi monkeys or other nonhuman primate species.

Our hypotheses concerning sex differences in OXTR binding density were largely unsupported. None of the comparisons between males’ and females’ OXTR binding in any of our regions of interest attained significance. These null findings are consistent with several other investigations in rodents showing no sex differences in hippocampal OXTR binding in many species, including C57BL/6 mice^22,43^, golden hamsters (*Mesocricetus auratus*)^42^, naked mole rats (*Heterocephalus glaber*)^44^, Wistar rats^45^, and prairie voles^47^. Although there are well-documented sex differences in hippocampal plasticity (for a comprehensive review, see^76^), the results of our study may suggest that these plasticity differences are unrelated to OXTR binding in titi monkeys. However, it is also possible that this study was underpowered to detect sex differences, and further research is needed. We note that even though none of the sex difference analyses attained formal statistical significance, exploratory analyses revealed trend-level sex differences in the dentate gyrus, with females exhibiting higher OXTR binding than males, and in CA2/3, with males exhibiting higher OXTR binding than females. In both cases, the sex differences were large (Cohen’s *d* > 1.0). We also note that despite the many rodent studies showing null findings (see above), some rodent studies have found sex differences in hippocampal OXTR binding^48–51^. Given these trend-level results and the small sample size in this study, further investigation is needed with a larger sample size to assess how OXTR binding differs between males and females, especially in primate species.

In this study we showed that OXTR binding in the titi monkey hippocampal formation differed between parents and non-parents and was associated with affiliative behavior with a pair mate. To our knowledge, this is the first investigation of hippocampal OXTR binding and social behavior in a nonhuman primate species. We found that OXTR binding in the presubiculum was higher in parents compared to non-parents and that high OXTR binding in the presubiculum was correlated with low levels of affiliative behavior. These findings may suggest that oxytocin acts in the presubiculum to contribute to socio-cognitive processes associated with bonding and attachment. We also report the first investigation of sex differences in hippocampal OXTR binding in a nonhuman primate species. While we found some evidence of a sex difference in the dentate gyrus and CA2/3, these comparisons were only significant at a trend level. One limitation of this study is that it was potentially underpowered. However, we note that the sample size (10 subjects) is nonetheless larger than many other investigations of central OXTR binding in human or non-human primates (see, for example^28,77,78^). Continued research is needed using larger sample sizes to investigate how oxytocin and OXTR binding contribute to the neurobiology of parenting and pair bonding and whether similar patterns of OXTR binding are found in other primate species.

## Methods

### Subjects and tissue

Subjects were 10 titi monkeys (ages 4–7.5 years old), including six parents (three males, three females) and four non-parents (two males, two females) (for a detailed summary of the subjects, see Supplementary Table S1). All procedures involving animals were reviewed and approved by the Animal Care and Use Committee at the University of California, Davis, and were in compliance with the National Institutes of Health guide for the care and use of Laboratory animals. All subjects were housed with an opposite sex pair mate, and the subjects that were parents were also housed with offspring. In all cases, subjects had been paired with their pair mate for at least 6 months at the time of death (average = 23 months, range 9–33 months), an adequate amount of time for a pair bond to form^79,80^. None of the subjects in this study were paired with each other. Subjects were considered parents if they had produced at least one surviving offspring (i.e., an offspring living more than 1 week) that they reared for at least 1 month (parents reared offspring between 2 and 14 months, median 4.4 months). In some cases, subjects that met criteria for the parent group had also previously produced a non-surviving offspring (*n* = 2; see Supplementary Table S1). Subjects that did not produce any offspring (*n* = 1) or that produced only non-surviving offspring (*n* = 3) were considered non-parents. To control for potential effects of pregnancy and parturition associated with producing a non-surviving offspring, a categorical variable indicating whether monkeys had ever produced a non-surviving offspring or not was tested as a covariate in analyses (see “Data analysis” section below). There were, on average, no differences between parents and non-parents in age at death, weight at death, or the amount of time paired with the pair mate (*p* > 0.38). Males and females did not differ in the amount of time paired with the pair mate (p = 0.42); however, males, on average, weighed more (*t*_(8)_ = − 3.43, *p* = 0.009, *d* = − 2.17) and were slightly older at the time of death (*t*_(8)_ = − 4.17, *p* = 0.003, *d* = − 2.64).

Brain tissue was collected opportunistically from subjects that were euthanized for health reasons, all of which were unrelated to this project and non-neurological. Brains were removed shortly after euthanasia and rinsed with a PBS solution, blocked coronally and by hemisphere, and then frozen at − 80℃. For sectioning, tissue was brought to − 20℃, sectioned with a cryostat in 20 μm coronal slices, and mounted on glass Fisher SuperFrost-plus slides. From the available bank of brain tissue, 3–8 representative sections spanning the hippocampus were selected per subject (yielding a total of 70 sections). Slides were stored in sealed slide boxes with a humidity sponge at − 80℃ until ready for further use.

### ^125^I-OVTA autoradiography and binding quantification

OXTR receptor autoradiography was completed using a previously established protocol^28^. Brain tissue sections were removed from – 80 ℃ storage and thawed for 1 h at room temperature, fixed lightly in 0.1% paraformaldehyde, washed twice in Tris Buffer, and then incubated with 50 pM of the radioligand ^125^I-ornithine vasotocin analog (OVTA; PerkinElmer, Waltham, MA, USA) for 1 h. A previous experiment from our lab demonstrated that this concentration of the ^125^I-OVTA radioligand is sufficient to detect OXTR binding in the titi monkey brain, and competitive binding experiments showed that this radioligand is specific to OXTR in titi monkeys^28^. Given these results, we did not repeat the competitive binding experiments in this study and proceeded with the optimized protocol based on that published study. After incubation, slides were exposed to BioMax MR film (Kodak, Rochester, NY, USA) for 3 days with a set of ten ^125^I standard microscales (American Radiolabeled Chemicals, St. Louis, MO, USA). All researchers received the necessary training and certification to safely handle the hazardous chemicals and radioactive substances used in this protocol.

Binding density was quantified using the MCID Core Digital Densitometry system (Cambridge, UK). Images of the film were loaded into the software using a light box and SPOT camera (Diagnostic Instruments, Sterling Heights, MI, USA) connected to a computer. First, a flat-field correction for luminosity levels was determined and then applied to all images. Next, optical binding values were determined for the ^125^I standards to generate a standard curve, which were used to determine and extrapolate binding density values for the following brain regions of interest: the hippocampus subfields CA1, CA2, CA3, and CA4, the dentate gyrus, the subiculum, presubiculum layer 1 (PSB1), and presubiculum layer 3 (PSB3). Boundaries for each ROI were based on previously defined boundaries for hippocampus^81,82^ and presubiculum subregions^54,56,83^ (see Fig. 1 for a schematic of the boundaries used for each ROI). Adjacent slides were counterstained for Nissl substance and used to guide the visual determination of all anatomical boundaries during quantification. Due to a lack of clear neuroanatomical boundaries on the film for CA2 and CA3, these subfields were quantified together and will be referred to as CA2/3. In addition to specific subregions described above, binding density was determined for the total hippocampal formation by tracing around CA1, CA2/3, CA4, the dentate gyrus, and the subiculum, and the total presubiculum binding density was quantified by tracing around PSB1 and PSB3. Although most sections were suitable for quantifying all brain regions of interest (*n* = 55), some sections were not fully quantified for all regions due to tissue damage affecting some of the targets (*n* = 15). For each region quantified, the measured OXTR binding density was normalized by subtracting the average background level, which was determined by measuring a white matter tract on the same section. After normalization, OXTR binding density was averaged across each subject for each brain region, resulting in nine values for each subject (one for each of the following brain regions: CA1, CA2/3, CA4, dentate gyrus, subiculum, PSB1, PSB3, total hippocampus, total presubiculum).

### Nissl staining

To visualize the brain regions quantified, several sections adjacent to those used for autoradiography were stained for Nissl substance using a protocol previously used in our lab see^84^, with slight modification. Sections were soaked in a 4% paraformaldehyde solution at 4 ℃ for 1 week, then defatted at room temperature in a chloroform-ethanol solution for 2 h. Next, sections were rehydrated in a series of ethanol solutions (ranging from 100 to 50% ethanol), and then were dipped for 20 s in thionin to stain for cell bodies. Using the same series of ethanol solutions, sections were then dehydrated, and then cleared in xylenes. Coverslips were then applied with a DPX mounting medium. Images of Nissl stains were taken with a light box and SPOT camera (Diagnostic Instruments, Sterling Heights, MI, USA).

### Partner affiliation observations

Partner affiliation was determined from longitudinal daily observations conducted approximately 5 days per week, every 2 h, resulting in 5–6 observations per day. Data were available for eight of the ten subjects, and all available affiliation data for each subject was used in analyses. Titi monkey pairs were marked as either *Tail Twining*, in *Contact*, or in *Proximity* (within one arm’s length) to each other, or *None*. For each affiliation state (*Proximity*, *Contact*, and *Tail Twining*), the total number of observations that the subject was observed in that state was divided by the total number of observations performed that day, and then each daily percent was averaged across days for each subject. Pair tenure (the amount of time that subjects had been paired together when data collection first began) varied between subjects (ranging from newly paired to 17 months already paired at the beginning of data collection, average = 8 months; see Supplementary Table S1). The total number of days that affiliation observations were conducted also varied between subjects (ranging from 5–21 months’ worth of data, average = 17 months’ worth). Data collection typically stopped when the subject died, the subject’s pair mate died, or if the pair was separated. Both pair tenure and the total amount of data collected were considered as covariates in analyses (see “Data analysis” section below). This approach was taken because a previous investigation from our lab using affiliation data from other titi monkey pairs in our colony found a correlation between pair tenure and the frequency of affiliative behaviors^80^, and because, in the current study, pair tenure was in some cases correlated with the average frequency of the observed affiliation states (see Table 1).

### Infant carrying observations

For the six subjects that were parents, infant carrying was determined from longitudinal daily observations conducted at the same frequency as the pair mate affiliation observations. Starting on the day that offspring were born, offspring were observed 5 days per week, five to six times a day, and marked as either on *Father*, on *Mother*, or *Off*. The amount of available observation data ranged from approximately 2–9 months’ worth of data (see Supplementary Table S1). In the case of one male parent that had two surviving offspring (see Supplementary Table S1), infant carrying data were used for the second offspring, as this offspring was the most recently parented. For analyses, the total number of times offspring were observed on the focal parent was divided by the total number of observations that day, and then averaged across days for each subject. Because the total amount of available data varied between subjects, infant carrying was also calculated for the first 30 days and the first 60 days of the infant’s life. However, because preliminary analyses with these variables yielded similar results as the variable calculated from all available infant carrying data, we report only the analyses using the average across all available data for each subject (the analyses using the first 30 days and the first 60 days are available upon request). The total amount of data collected was not controlled for in analyses because it was not correlated with average infant carrying (*r* = − 0.15, *p* = 0.78).

### Data analysis

All analyses were performed in R programming^85^. To test whether average OXTR binding density differed by sex or parental status, MANOVAs with Pillai’s trace were used. MANOVA analyses were performed separately for two blocks of dependent variables: OXTR binding density in hippocampus subregions (CA1, CA2/3, CA4, dentate gyrus, and subiculum) and OXTR binding density in presubiculum subregions (PSB1 and PSB3). In each analysis block, either sex or parental status was used as the independent variable, yielding a total of four MANOVA that were performed. Although the interaction between sex and parental status was assessed in preliminary analyses, the interaction was not significant for either block of dependent variables (likely due to inadequate power), and consequently these results are not reported in the main text (see Supplementary Table S2). For each MANOVA with a significant main effect, post-hoc t tests were performed to assess sex differences or parental status differences in OXTR binding density in each brain region analyzed in the block of dependent variables. To assess whether any specific patterns across the hippocampus or presubiculum subregions could be generalized to the total binding across the hippocampus or presubiculum, t tests were also performed with the OXTR binding across the total hippocampus or the total presubiculum as the dependent variable, and parental status or sex as the dependent variable. For all t tests, Cohen’s *d* effect size was calculated. To control for possible confounding factors, MANOVA with significant effects were also repeated once with age at death, once with weight at death, and once with a categorical variable indicating whether the subject had ever produced a non-surviving offspring included in the model. Because none of these covariates attained significance in any model tested, the results of these models are not reported (they are available upon request). Because Kolmogorov–Smirnov tests showed all variables were normally distributed, data were not transformed for analysis.

To assess whether average OXTR binding was associated with average partner affiliation states, Pearson correlation analyses were performed for each average partner affiliation state (*Proximity*, *Contact*, and *Tail Twining*) and OXTR binding density in each of the subregions quantified, as well as in the total hippocampus and total presubiculum. Follow up analyses were performed using Spearman rho rank correlation, a non-parametric analysis. This approach was taken because the average partner affiliation states were percentages and were therefore bound between a range of 0 and 1. Further follow-up analyses were performed using multiple regression to control for pair tenure, the total amount of affiliation data collected, and the age at death, possible confounding factors that were in some cases associated with average affiliation states (see Table 1). Because age at death and the total amount of partner affiliation data collected were not significant covariates in any analysis and were not significantly associated with average affiliation states, these analyses are not reported (they are available upon request).

To assess associations between average infant carrying and average OXTR binding density in each brain region, Pearson correlation and Spearman rho analyses were used.

## Supplementary information

Supplementary Tables

## Data Availability

The data analyzed in this study will be made available to any qualified researcher upon reasonable request.
