# Supplementary material for: Oxytocin receptor binding in the titi monkey hippocampal formation is associated with parental status and partner affiliation
Source: Sci Rep. 2020 Oct 14;10:17301. doi: 10.1038/s41598-020-74243-1 (PMC7560868; doi:10.1038/s41598-020-74243-1)
Supplement: Supplementary file 1 — Supplementary Tables [file 41598_2020_74243_MOESM1_ESM.docx]

**Supplementary Material**

Oxytocin Receptor Binding in the Titi Monkey Hippocampal Formation Is Associated with Parental Status and Partner Affiliation

Baxter, A.*^1,2^; Anderson, M.^1^; Seelke, A.M.^1,2^; Kinnally, E.L.^1,2^; Freeman, S.M.^3^; Bales, K.L.^1,2,4^

1) California National Primate Research Center

2) University of California, Davis, Department of Psychology

3) Utah State University, Department of Biology

4) University of California, Davis, Department of Neurobiology, Physiology, and Behavior

|  |  |  | Variables Related to Parental Status and Infant Carrying Data Collection | | | | | | Variables Related to the Pair Mate and Affiliation Data Collection | | | | |
| --- | --- | --- | --- | --- | --- | --- | --- | --- | --- | --- | --- | --- | --- |
| Subject | Parent Status | Sex | Number of Offspring Born | | Number of Offspring that Survived | | Amount of Infant Carry Data Collected (Months) | Surviving Offspring Age When Subject Died (Months) | Age when Paired with Partner (Years) | Age when Affiliation Data was First Collected (Years) | Age at Death (Years) | Amount of Time with Partner (Years): | Amount of Affiliation Data Collected (Years) |
| 01 | Non-Parent | F | 1 | 0 | | - | | - | 4.2 | 4.2 | 4.9 | 0.8 | 0.7 |
| 02 | Non-Parent | F | 2 | 0 | | - | | - | 1.8 | 3.1 | 4.4 | 2.6 | 1.8 |
| 03 | Non-Parent | M | 1 | 0 | | - | | - | 5.4 | 5.6 | 7.0 | 1.5 | 1.8 |
| 04 | Non-Parent | M | 0 | 0 | | - | | - | 2.8 | - | 5.4 | 1.0 | - |
| 05 | Parent | F | 1 | 1 | | 5 | | 5* | 1.5 | 3.0 | 4.3 | 2.7 | 1.8 |
| 06 | Parent | F | 1 | 1 | | 4 | | 4 | 2.2 | 3.1 | 4.3 | 2.1 | 1.8 |
| 07 | Parent | F | 2 | 1 | | 9 | | 9* | 2.6 | - | 4.0 | 1.5 | - |
| 08 | Parent | M | 1 | 1 | | 2 | | 2 | 4.4 | 4.7 | 5.2 | 0.8 | 0.4 |
| 09 | Parent | M | 2 | 1 | | 4 | | 4 | 4.3 | 5.6 | 6.9 | 2.7 | 1.8 |
| 10 | Parent | M | 2 | 2 | | 5, 4 | | 14, 4 | 5.7 | 5.7 | 7.3 | 1.6 | 1.6 |

Supplementary Table S1. Summary of Subjects Used in Analyses.

The table shows a summary of the subjects used in the analyses. Subjects were considered parents if they had a surviving offspring (i.e., the offspring lived more than one week) that they reared for at least one month of life. All subjects were euthanized while they were housed with their partner and offspring, except where indicated (see footnotes below). In cases where the offspring died before the subject did, the Surviving Offspring Age When Parent Died indicates the infant’s age when it died (these instances are labeled with an *).

**Abbreviations**: F indicates female, M indicates male, - indicates no data

| MANOVA Model | *DF*_Between_ | *DF*_Within_ | Pillai’s Trace | *F* | *p* |
| --- | --- | --- | --- | --- | --- |
| ***Hippocampus Regions*** |  |  |  |  |  |
| Parental Status | 1 | 6 | 0.52 | 0.43 | .81 |
| Sex | 1 | 6 | 0.90 | 3.55 | .23 |
| Sex by Parental Status | 1 | 6 | 0.62 | 0.66 | .69 |
| ***Subiculum Regions*** |  |  |  |  |  |
| Parental Status | 1 | 6 | 0.76 | 8.01 | .028 |
| Sex | 1 | 6 | 0.50 | 2.52 | .18 |
| Sex by Parental Status | 1 | 6 | 0.22 | 0.69 | .54 |

Supplementary Table S2. MANOVA Models Assessing Sex by Parental Status Interactions.

The table shows the results of preliminary MANOVA analyses assessing the interaction between Parental Status and Sex on OXTR binding across hippocampus subregions (CA1, CA2/3, CA4, dentate gyrus, and subiculum) and presubiculum regions (PSB1 and PSB3). Because of small cell sizes, these analyses are likely underpowered to detect interaction effects. Although none of the interaction terms were significant in these analyses, the results of the main effects approximate the results presented in the main text, in which each MANOVA was performed separately for parental status and sex.
